# Development of human electrophysiological brain networks

**DOI:** 10.1152/jn.00293.2018

**Published:** 2018-10-24

**Authors:** Paul M. Briley, Elizabeth B. Liddle, Madeleine J. Groom, Helen J. F. Smith, Peter G. Morris, Giles L. Colclough, Matthew J. Brookes, Peter F. Liddle

**Affiliations:** ^1^Centre for Translational Neuroimaging in Mental Health, Institute of Mental Health, School of Medicine, University of Nottingham, Nottingham, United Kingdom; ^2^Sir Peter Mansfield Imaging Centre, School of Physics and Astronomy, University of Nottingham, Nottingham, United Kingdom; ^3^Oxford Centre for Human Brain Activity, Wellcome Centre for Integrative Neuroimaging, Department of Psychiatry, University of Oxford, Oxford, United Kingdom

**Keywords:** default mode network, development, frontoparietal network, magnetoencephalography, salience network

## Abstract

Functional activity in the human brain is intrinsically organized into independently active, connected brain regions. These networks include sensorimotor systems, as well as higher-order cognitive networks such as the default mode network (DMN), which dominates activity when the brain is at rest, and the frontoparietal (FPN) and salience (SN) networks, which are often engaged during demanding tasks. Evidence from functional magnetic resonance imaging (fMRI) suggests that although sensory systems are mature by the end of childhood, the integrity of the FPN and SN develops throughout adolescence. There has been little work to corroborate these findings with electrophysiology. Using magnetoencephalography (MEG) recordings of 48 participants (aged 9–25 yr) at rest, we find that beta-band functional connectivity within the FPN, SN, and DMN continues to increase through adolescence, whereas connectivity in the visual system is mature by late childhood. In contrast to fMRI results, but replicating the MEG findings of Schäfer et al. (Schäfer CB, Morgan BR, Ye AX, Taylor MJ, Doesburg SM. *Hum Brain Mapp* 35: 5249–5261, 2014), we also see that connectivity between networks increases rather than decreases with age. This suggests that the development of coordinated beta-band oscillations within and between higher-order cognitive networks through adolescence might contribute to the developing abilities of adolescents to focus their attention and coordinate diverse aspects of mental activity.

**NEW & NOTEWORTHY** Using magnetoencephalography to assess beta frequency oscillations, we show that functional connectivity within higher-order cognitive networks increases from childhood, reaching adult values by age 20 yr. In contrast, connectivity within a primary sensory (visual) network reaches adult values by age 14 yr. In contrast to functional MRI findings, connectivity between cognitive networks matures at a rate similar to within-network connectivity, suggesting that coordination of beta oscillations both within and between networks is associated with maturation of cognitive skills.

## INTRODUCTION

### 

#### Resting-state networks measured with fMRI.

A robust set of functionally connected “resting-state” brain networks has been one of the most important discoveries in functional magnetic resonance imaging (fMRI) over the last two decades. Fluctuations in fMRI blood oxygenation level-dependent (BOLD) signal over time, assumed to reflect neural activity, are correlated between brain regions. These correlated regions form the nodes of multiple distinct networks that exhibit consistency in spatial configuration across individuals. Moreover, networks observed during rest are very similar to those observed during the performance of tasks (Toro et al. 2008).

Fox et al. (2005) showed that a network of “task-positive” brain regions that show increased BOLD signal in response to cognitively demanding tasks is anticorrelated at rest with a network of “task-negative” regions that show decreased BOLD signal, a network known as the default mode network (DMN). Furthermore, Seeley et al. (2007) showed that the task-positive regions form two dissociable networks: a frontoparietal network (FPN) implicated in executive control and a salience network (SN), also known as the cingulo-opercular network, involved in salience processing. In contrast, the DMN is especially prominent during rest and has been hypothesized to reflect internally directed mental activity (Fransson 2005; Mason et al. 2007), although its role has been a topic of debate (Gilbert et al. 2007; Raichle and Snyder 2007). The anticorrelation between the DMN and task-positive regions found by Fox et al. (2005) is observed after covarying for global BOLD fluctuations. If the effect of global fluctuations is not removed, the correlations between DMN and task-positive sites are typically near zero (Fox et al. 2009), whereas within-network correlations are positive. Notably, both during task and during rest there is clear segregation between DMN sites and task-positive sites in FPN and SN.

#### Development of fMRI resting-state networks.

Although there is consistent evidence that these networks are spatially and functionally distinct in adulthood, fMRI evidence suggests that they emerge gradually over the course of childhood and adolescence. Fair et al. (2009) reported that as age increases from childhood to adulthood there is a shift from predominantly local connections between brain regions to long-range connections. In particular, they found that with increasing age the strength of connections within the FPN, SN, and DMN increased. However, rigorous reduction of effects attributable to head movement indicates that these findings are partially confounded by spuriously strong local connectivity and underdetection of long-range connectivity in younger children resulting from greater movement artifacts (Power et al. 2012). Nonetheless, even after allowing for movement artifacts, developmental changes in the connectivity between networks are discernible.

In a resting-state fMRI study of 6- to 8-yr-old children, de Bie et al. (2012) found that the DMN and other resting-state networks involved in higher-order cognitive functions were fragmented and incomplete, reflecting less developed functional connectivity. In a resting-state fMRI study of 10- to 26-yr-olds, Marek et al. (2015) found that although within-network connectivity had stabilized before adolescence, integration between networks increased during adolescence. This increase was associated with increased ability to inhibit errors in an antisaccade task, indicating a relationship between network integration and maturation of cognitive control.

#### Development of electrophysiological resting-state networks.

Although the BOLD signal provides an index of the changes in blood flow required to meet the metabolic demands of active neurons and glia, it is only an indirect measure of neural activity. Electroencephalography (EEG) and magnetoencephalography (MEG) provide a more direct measurement of neural signals, with sampling rates on the timescale of neural transmission, albeit with lower spatial resolution. MEG studies have discovered networks with spatial distributions similar to the BOLD-defined resting-state networks (Brookes et al. 2011c; Hipp et al. 2012). These electrophysiological networks are revealed by between-node correlations in the amplitude envelopes of neural oscillations in specific frequency bands. In most of the networks, these correlations are strongest in the beta band (≈13–30 Hz), although between some nodes of the DMN both alpha (≈8–13 Hz) and beta oscillations exhibit strongly correlated activity (Brookes et al. 2011c).

Using MEG, Schäfer et al. (2014) investigated the development of seven resting-state networks (including the FPN and DMN) between ages 6 and 34 yr. They reported a linear increase in connectivity in the alpha and beta bands within these networks over that age range. Furthermore, in contrast to Fair et al.’s (2009) BOLD findings, namely, that the networks become more clearly segregated with age, Schäfer et al. (2014) reported that in MEG between-network amplitude envelope correlations strengthened with age. However, they tested only for linear dependence on age across the entire age range and did not investigate whether within- and between-network connectivity develop at different rates at different developmental stages or whether the developmental trajectory of within-network connectivity differs between networks.

We have previously reported (Brookes et al. 2018) results from an MEG study of children and young people aged 9–25 yr showing that stationary connectivity between brain regions defined with the AAL atlas (Tzourio-Mazoyer et al. 2002), averaged across the whole brain, increased with age in the alpha and beta frequency bands. We also found that the temporal stability of transient dynamic spatio-temporal brain states increased with age. In view of the fMRI findings of de Bie et al. (2012) that the DMN and other resting-state networks involved in higher-order cognitive functions were fragmented and incomplete in 6- to 8-yr-old children, together with evidence from cognitive studies that executive functions mature more slowly than simple perceptual functions (Fuster 2002), in this study we hypothesized that connectivity in the three networks implicated in higher-order cognitive functions, namely, the DMN, FPN, and SN, would mature more slowly than the connectivity of primary sensory networks, for example, the visual network that embraces primary and extrastriate visual cortex.

#### Differences between fMRI and electrophysiological resting-state networks.

The relationship between the BOLD signal and the beta-band MEG signal has yet to be established. In contrast to the BOLD signal, in which task-related changes in the “task-negative” DMN are in the opposite direction to those in the “task-positive” FPN and SN, widespread beta desynchronization in all three networks is observed during performance of cognitively demanding tasks. For example, during the *n*-back working memory task, which demands the simultaneous maintenance and processing of information, beta desynchronization is widespread, especially in the midline and lateral frontal and parietal regions involved in the FPN, SN, and DMN (Brookes et al. 2011b). Moreover, using simultaneous EEG-fMRI, Mantini et al. (2007) found that although global beta power was negatively correlated with BOLD in regions of task-positive networks, it was positively correlated with BOLD in DMN regions. These findings have potentially major implications for understanding the relationship between oscillatory activity observed with MEG and the BOLD signal observed with fMRI, and for understanding the relationship between BOLD activity and neural activity.

Consistent with the sizable between-network correlations seen in MEG by Schäfer et al. (2014), Tewarie et al. (2014) found that alpha- and beta-band functional connectivity is strongest between nodes that form a pattern resembling that of the “structural core” identified by Hagmann et al. (2008) based on the density of white matter connections between regions. This core embraced medial and lateral parietal cortex and extended into lateral temporal cortex and to medial frontal cortex. Hagmann et al. (2008) demonstrated similarity between this structural core and a functional core based on correlations between resting-state BOLD signals. This finding suggests that the connections within the structural core may serve to integrate the functions of distinguishable resting-state networks. In a later study, Hagmann et al. (2010) demonstrated that the white matter connections of the structural core continue to develop through adolescence into young adulthood. It is thus plausible that alpha and beta oscillations are also engaged in both within- and between-network communication and that this communication continues to strengthen through adolescence.

However, when investigating network connectivity with MEG, an important caveat must be borne in mind. There is no unique solution to the inverse problem of identifying the location of the source of MEG signals detected with sensors outside the brain. Techniques such as beamforming, employed by Brookes et al. (2011c) to delineate resting-state networks in MEG and by Schäfer et al. (2014) to investigate their developmental trajectory, constrain the solution of the inverse problem to minimize the contribution of sources in extraneous brain regions to the estimated strength of the sources at a location of interest. Whereas MEG has resolution of order 5–8 mm or even less when identifying a strong local source, in situations where diffuse sources from many brain regions contribute to the observed signal the signals from different sources can be difficult to distinguish because of inaccurate forward modeling that can generate the mislocalization of sources and also because of blurring inherent in the inverse problem. This is the problem of signal leakage, which could potentially account for at least some of the positive correlations between network nodes. Schäfer et al. (2014) minimized the risk of leakage by assessing connectivity between regions separated by at least 35 mm. Another approach to the problem of leakage is to remove zero-time lag correlations between the location of interest and other regions likely to be contributing to the observed signal.

#### Aims of the present study.

Using MEG data from the same sample of children and young people as reported in Brookes et al. (2018), we set out to investigate the maturation of beta-band correlations between the nodes of the DMN, FPN, SN, and a visual network (VN) embracing primary and extrastriate visual cortex between the ages of 9 and 25 yr. We focused on beta-band recordings because this is the frequency band within which most resting-state networks are clearly expressed (Brookes et al. 2011c). To control for spurious positive correlations introduced by leakage, we employed a symmetrical orthogonalization developed by Colclough et al. (2015). This procedure removes all shared zero-time lag correlations between all regions in the analysis. We also used estimates of the lead fields at each sensor (the predicted signals arising from a unit source at each source location) to control for relative differences in signal strength attributable to differences in head size and shape, and thus distance from the signal sensors.

We made the following predictions: *1*) With increasing age, positive correlations in the amplitude of beta oscillations will increase between nodes of the DMN, FPN, and SN. *2*) Crucially, the increase in correlation strength between the nodes of each of the three attentional networks (DMN, FPN, and SN) will follow a trajectory similar to the correlations within these networks. This is in stark contrast to the anticorrelation (or lack of correlation) between the DMN and the FPN/SN seen in fMRI studies. *3*) The correlations of beta oscillations within and between attentional networks will mature more slowly than the correlations between nodes of the VN.

**Fig. 1.**
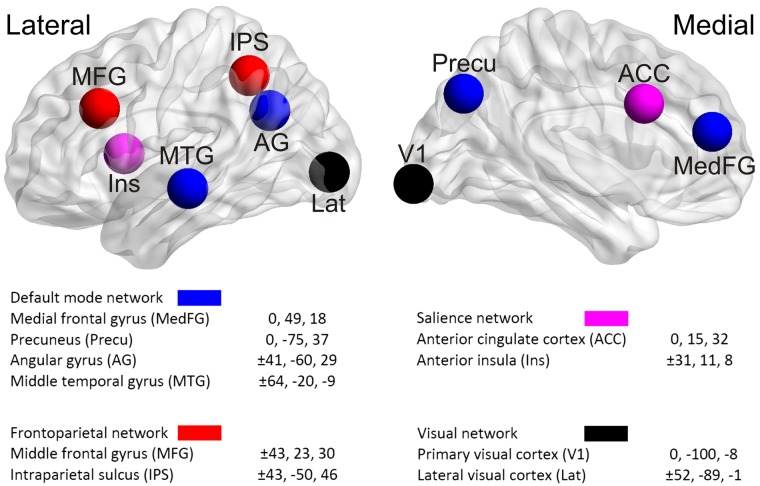
The spherical regions of interest (ROIs) that represent the nodes of the 4 networks, displayed using BrainNet Viewer (http://www.nitrc.org/projects/bnv/) (Xia et al. 2013) with the ICBM-152 template brain (Mazziotta et al. 2001b). The ROIs are located symmetrically in the 2 hemispheres. The coordinates of the center of each ROI are specified in Montreal Neurological Institute space according to loci defined in the functional atlas developed by Yeo et al. For each participant, the centers of the ROIs were mapped onto the corresponding location in that individual’s brain as described in materials and methods.

The locations of the nodes of the networks are depicted in Fig. 1.

## MATERIALS AND METHODS

### 

#### Participants.

MEG measurements were made in 51 healthy participants (27 female, 24 male) between the ages of 9 and 25 yr, inclusive. Exclusion criteria included a history of a neurological disorder or a contraindication to MRI scanning (such as metal in the body). Three participants were excluded because of excessive head movement (ages 9, 11, and 18 yr). For some analyses, the remaining 48 participants were split into three groups: a child group (under 13 yr; 9 participants: 5 female, 4 male), an adolescent group (13–18 yr; 11 participants: 7 female, 4 male), and a young adult group (19 yr or older; 28 participants: 15 female, 13 male). Participants (16 yr and over) or a parent (of those under 16 yr) provided written informed consent. The study was approved by the Ethics Committee of the University of Nottingham Faculty of Medicine and Health Sciences.

#### MRI acquisition.

A T1-weighted structural MRI scan was acquired for each participant with an MPRAGE sequence on a Phillips Achieva 3T system (volume transmit and 32-channel receive head coil; 1-mm isotropic voxels; TE 8 ms, TI 960 ms, TR 3s, FA 8°).

#### MEG acquisition.

A 275-channel CTF MEG system was used in its third-order synthetic gradiometer configuration to record 5 min of awake resting-state data at a sampling rate of 600 Hz. During acquisition, participants lay supine and were instructed to fixate on a centrally presented marker that was back-projected onto a screen 35 cm ahead of them. Head padding, or a glass fiber insert for the MEG helmet for participants with smaller heads, was used to reduce movement and to center the head equidistant from all gradiometer coils. Additional data (not presented here) were recorded during cognitive tasks in separate runs. Before recording, a head shape was obtained for the participant with a three-dimensional digitizer (Polhemus); the locations of three head position coils were also digitized (nasion and left/right preauricular points), and these were used to track changes in head position throughout the MEG recordings. A head shape extracted from the participant’s structural MRI was coregistered with the head shape from the three-dimensional digitizer to enable accurate calculation of MEG lead fields.

#### MEG preprocessing including removal of segments contaminated by head movement.

The 5-min resting-state recordings were segmented into 10-s epochs. Given concerns in the fMRI literature that head movement can artifactually reduce measurements of long-range brain connectivity (Power et al. 2012), we excluded all epochs that had a head position coil >4 mm from its starting position at any time point. Despite this strict criterion, 41 participants lost no epochs. In the three participants who were excluded from the analysis, more than half (i.e., >15) of the epochs were lost because of head movement. Epochs with excessive noise were then identified through visual inspection (2 epochs were lost for 2 participants; 3 epochs were lost for 1 participant). Data were frequency-filtered into the beta (13–30 Hz) and alpha (8–13 Hz) bands for further analyses.

#### Regions of interest.

A spatial transform from each participant’s coregistered structural MRI to the standard Montreal Neurological Institute (MNI)152 brain (Mazziotta et al. 2001a) was determined with SPM 8 (https://www.fil.ion.ucl.ac.uk/spm). This also produced the inverse transform, which was used to transform regions of interest (ROIs) defined in MNI space into equivalent regions of the coregistered structural MRI, which was in the space of the MEG system. The ROIs themselves were defined as spheres of radius 10 mm centered on MNI coordinates identified by Yeo et al. (2011) as nodes of resting-state networks in a large fMRI data set (incorporating a total of 1,000 participants). Sixteen nodes were chosen, to represent the DMN, FPN, SN, and a visual sensory network (see Fig. 1). In the case of 14 nodes, specific MNI coordinates specified by Yeo et al. (2011) were employed. In the cases of two of the nodes (precuneus and dorsolateral prefrontal cortex) where the coordinates specified by Yeo et al. did not correspond closely to the coordinates of nodes employed by Fair et al. (2009), coordinates specified by Fair et al. were employed after transformation from Talairach to MNI coordinates. In both instances, the selected site nonetheless lay within the relevant region defined within the Yeo et al. atlas. For each participant, the spheres were transformed from MNI space into equivalent regions of the participant’s brain and the transformed ROIs were used for that participant in all subsequent analyses. The transformed ROIs were masked with a downsampled brain mask to exclude nonbrain voxels and to convert the ROIs to 8-mm^3^ voxel size.

#### Deriving an activity time course for each ROI.

For each participant, the time course of activity for each voxel in the grid of 8-mm^3^ voxels spanning the brain was calculated with a scalar linearly constrained minimum-variance beamformer applied to the MEG data (Brookes et al. 2011a; Robinson and Vrba 1999), with lead fields created with a multiple local sphere head model (Huang et al. 1999) and a dipole approximation of activity within each voxel (Sarvas 1987). Between 2,737 and 4,233 voxels were used for beamforming per participant [mean 3,455 voxels (SD 333)]. Dipole orientation was determined for each voxel by searching for the orientation that gave maximum signal-to-noise ratio. The covariance matrix was calculated over the full time series in the frequency band 13–30 Hz for the beta-band analyses and in the frequency band 8–13 Hz for the alpha-band analyses and then regularized with the Tikhonov method using a regularization value of 4 times the minimum eigenvalue of the unregularized covariance matrix, as used in Brookes et al. (2011c). For each voxel, the beamformer calculates a vector of weights, one for each sensor, to weight the measured magnetic fields at that sensor. This gives a time course of activity for each voxel. The beamformer weights are chosen to pass activity within the target voxel while maximally suppressing activity at nontarget regions (including external interference). Beamformer methods (at least, without leakage correction) would be expected to produce artifactually large connectivities between ROIs if the beamformer weights for the ROIs are highly correlated. In the present case, beamformer weight correlations, averaged across participants and calculated using the centroid voxel for each ROI, were <0.2 in all but three instances: between the right medial frontal gyrus and the right insula (*r* = 0.29), between the anterior cingulate cortex and the left insula (*r* = 0.30), and between the left medial frontal gyrus and the left insula (*r* = 0.46). The anterior cingulate cortex and left and right insula, considered part of the SN, are deeper brain regions to which MEG is less sensitive; therefore, as noted in discussion, effects involving the SN should be treated with caution. Principal component analysis was performed on the time series data for all voxels within each ROI. The first principal component was taken as a representative time course of activity for that ROI.

#### Reduction of signal leakage between ROI time courses.

The symmetrical multivariate leakage correction developed by Colclough et al. (2015) was applied to the 16 ROI time courses for each participant separately. This procedure removes all correlations at zero phase lag between ROI time courses by obtaining a set of orthonormal time courses that are closest to the data and then adjusting the orientations and lengths of the orthogonal vectors so that the corrected time courses are as close to the uncorrected time courses as possible. The technique removes any correlations attributable to signal leakage, at the expense of genuine zero-phase lag connectivity. For each ROI, the amplitude envelopes of the corrected time courses were then calculated as the absolute values of the Hilbert-transformed corrected time courses, and the amplitude envelopes were low-pass filtered at 1 Hz.

#### Calculating connectivity and adjusting for variation in head size and shape across participants.

The Pearson correlation coefficient was calculated between the amplitude envelope time courses for each pair of ROIs for each participant and then Fisher-transformed to give our measure of connectivity. This measure of connectivity is one of the most repeatable and reliable connectivity measures available in MEG (Colclough et al. 2016). In a developmental study, one important issue to address is the possibility that there might be appreciable differences in head size and shape across the age range. Connectivity values were therefore adjusted by multiple linear regression to allow for variation in MEG sensitivity across participants due to variation in head size and shape. An estimate of the sensitivity of MEG to a given ROI in a given participant was obtained by computing the Frobenius norm of the lead field vector for each voxel in that ROI and participant and then averaging the Frobenius norms across voxels in that ROI. The lead field vector for a voxel gives the signal that would be produced at each of the MEG sensors by a dipole source of unit strength located at that voxel. It is calculated using a participant’s anatomical MRI scan and thus is determined by the participant’s head size and shape, in particular the distance between each voxel and the MEG sensors. The Frobenius norm is the square root of the sum of squares of the values in a lead field vector.

For each pair of ROIs separately, the connectivity values, *C_j_*, across participants were modeled with *Eq. 1*:

(1) $$C_{j}={\text{α}}_{1}+{\text{α}}_{2}\left[1-e^{-{\text{α}}_{3}\left({\text{age}}_{j}-{\text{age}}_{0}\right)}\right]+{\text{α}}_{4}\cdot \Delta F1_{j}+{\text{α}}_{5}\cdot \Delta F2_{j}+{\text{ε}}_{j}$$

The equation incorporates estimates of differences in observed connectivity due to differences in Frobenius norm across participants. Δ*F*1*_j_* and Δ*F*2*_j_* are the differences between the Frobenius norms for participant *j* and the average across all participants for the two ROIs. The other terms are age*_j_* (age of participant *j*) and age_0_ (age of the youngest participant), an intercept term, α_1_; a term describing changes in connectivity with age, ${\text{α}}_{2}\left[1-e^{-{\text{α}}_{3}\left({\text{age}}_{j}-{\text{age}}_{0}\right)}\right]$; and a residual term, ε*_j_*. The term describing the change in connectivity with age represents a growth curve in which connectivity initially increases at a rate specified by α_3_ while the rate of increase gradually decreases asymptotically toward a value of 0 in adulthood. α_2_ represents the increase in connectivity from the earliest observed age to the asymptotic adult value. (The shape of the growth curve is illustrated in Fig. 4). α_1_, α_2_, α_3_, α_4_, and α_5_ are fitted parameters. After fitting, the estimated pairwise connectivity for the two ROIs for participant *j*, *C*_*j*_^′^, was obtained by subtracting out the two Frobenius norm terms (*Eq. 2*):

(2) $${C\prime }_{j}=C_{j}-{\text{α}}_{4}\cdot \Delta F1_{j}-{\text{α}}_{5}\cdot \Delta F2_{j}$$

In the VN, the adjustment had virtually no effect. This was likely because participants were scanned in a supine position—head size would have had minimal effect on distance from the visual cortex to the nearest (occipital) sensors in this position. The effect was small but appreciable in the DMN and FPN, likely since in individuals with larger heads the frontal cortex would have been closer to the nearest (frontal) sensors than in individuals with smaller heads (again because participants were scanned in the supine position). The correction led to an increase in estimated connectivity in the FPN and DMN in the younger individuals, thereby diminishing the estimated increase in connectivity with age. In the SN, the effects were small. The corrected connectivity values are used throughout this report.

## RESULTS

**Fig. 2.**
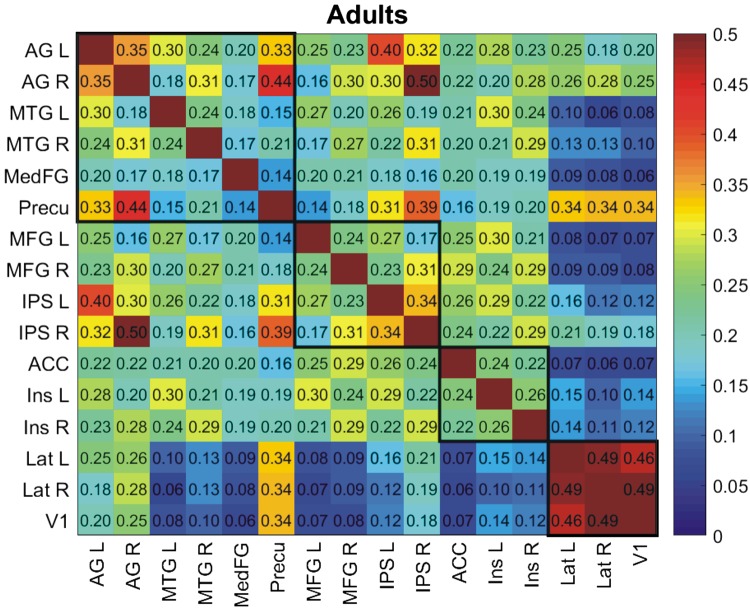
Mean connectivity in the beta band in the young adult group (age > 18 yr). Black boxes denote the 3 attentional networks and the visual network. Deep blue denotes zero connectivity; no negative correlations were observed. Connectivity is the Fisher-transformed correlation between amplitude envelopes of 13- to 30-Hz signals, with adjustment to allow for variation between participants in head shape and size. Abbreviations of region names are defined as shown in Fig. 1.

Figure 2 shows mean connectivity between each pair of ROIs for the adult group in the beta (13–30 Hz) frequency band. Black squares enclose the within-network pairs of DMN, FPN, SN, and VN nodes. Note that no negative connectivity values are apparent, indicating that no pair of ROIs has anticorrelated time courses. However, some values are relatively small, in particular those between VN nodes and nodes from other networks. Connectivity is strongest within the VN (extrastriate cortex left/right and a midline V1 ROI), with values from 0.46 to 0.49. In contrast, connectivity values between the VN and other network nodes apart from the precuneus are small. Connectivity between ROIs in the DMN is also particularly strong, although there are also sizable connectivities between some DMN and FPN ROIs. Relatively strong connectivity is typically seen for homologous pairs of ROIs in left and right cortex.

### 

#### Group differences in within-network connectivity.

**Fig. 3.**
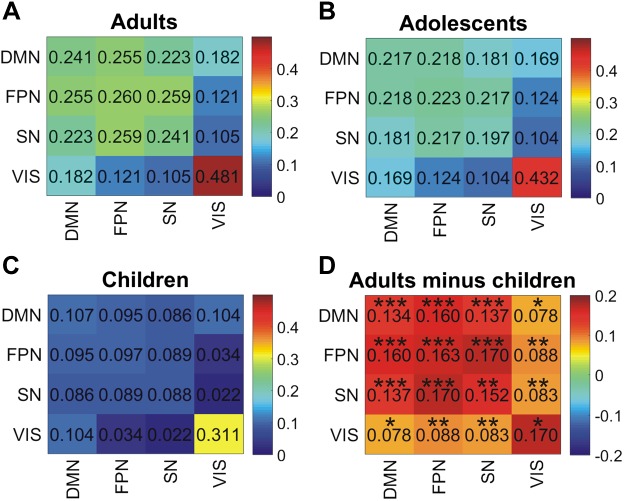
*A–C*: beta-band connectivities averaged across the pairs of regions of interest (ROIs) within each of the 4 networks in adults, adolescents (aged 13–18 yr), and children. *D*: difference in connectivity between adults and children. Uncorrected *P* values: *P < 0.05, **P < 0.005, ***P < 0.001. All cells marked ** or *** are significant at the corrected threshold (Bonferroni correction for the 11 comparisons). DMN, default mode network; FPN, frontoparietal network; SN, salience network; VIS, visual network.

Figure 3 shows connectivity within and between each network, averaging across ROI pairs, in each of the three age groups. This again shows the strong within-network and weak between-network connectivity for the visual region, in contrast to the strong within- and between-network connectivity for the DMN, FPN, and SN in the adult group. There are similar but weaker connectivities in the adolescent group and markedly weaker connectivities in the child group. The VN is the only network that is already well connected in children. We tested for fundamental developmental changes in connectivity by comparing the networks measured in adults and children, shown in Fig. 3*D*. All differences between groups have an uncorrected *P* value of <0.05, estimated with planned permutation tests, in which group membership was randomly reassigned, and repeated 10,000 times to form a null distribution of differences. The difference in within-network connectivity for the VN as well as the difference in connectivity between the VN and the DMN are nonsignificant when Bonferroni correcting for the 11 comparisons (i.e., using *P* < 0.0045), whereas all other differences remain significant.

Based on previous reports of strong within-network connectivity in the alpha band for the DMN (Brookes et al. 2011c), we also calculated differences in alpha-band (8–13 Hz) connectivity between adults and children for this network. Mean within-network connectivity for the DMN was 0.140 for adults and 0.059 for children, and this difference was significant at the *P* = 0.002 level with a planned permutation test (and significant at the Bonferroni-adjusted threshold of *P* < 0.0045).

#### Within-network connectivity vs. age.

**Fig. 4.**
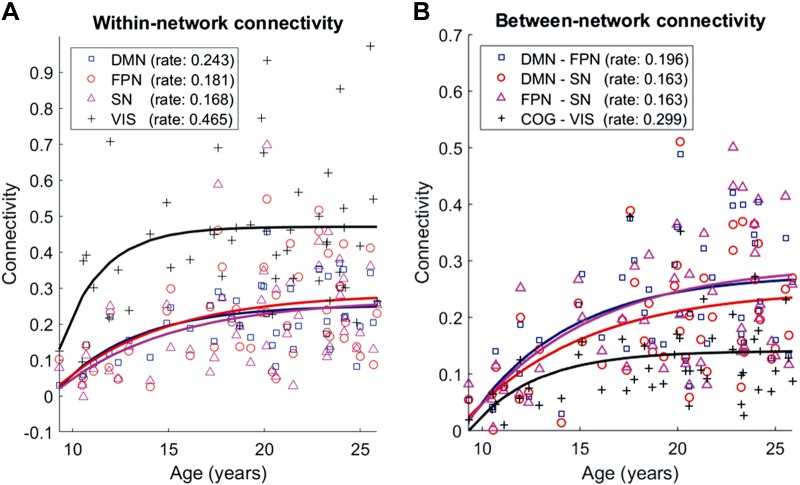
*A*: within-network beta-band connectivity for each of our 4 networks as a function of age. Symbols show individual participants, and lines show model fits. DMN, default mode network; FPN, frontoparietal network; SN, salience network; VIS, visual network. Fitted values of the parameter governing the initial rate of increase of connectivity with age (α_3_, which has units years^−1^) are given in the key. *B*: beta-band connectivity between each pair of cognitive networks as a function of age (DMN-FPN, DMN-SN, FPN-SN) and the mean connectivity between the 3 cognitive networks and the visual network as a function of age (COG-VIS).

Figure 4*A* plots within-network beta-band connectivity against age for all 48 participants for the four attentional networks. As described in materials and methods, we fitted the 48 data points for each network with a monotonic curve of the form (*Eq. 3*)

(3) $$C={\text{α}}_{1}+{\text{α}}_{2}\left[1-e^{-{\text{α}}_{3}\left(\text{age}-{\text{age}}_{0}\right)}\right]$$

where age_0_ is the age of the youngest participant; α_1_, α_2_, and α_3_ are fitted parameters. α_1_ represents the connectivity at the earliest observed age, α_2_ represents the increase in connectivity from the earliest observed age to the asymptotic adult value, and α_3_ represents the initial rate of increase in connectivity with age. *C* is the connectivity between pairs of ROIs after correction for between-subject variation in Frobenius norms, which reflect variation due to change in head size and shape with age (see materials and methods).

The model fit was significantly better than that of an intercept-only model, determined with *F*-tests comparing the difference in the residual sum-of-squares error between the full model and the intercept-only model, DMN: *F*(2,45) = 11.456, *P* < 0.001; FPN: *F*(2,45) = 6.110, *P* = 0.005; SN: *F*(2,45) = 4.783, *P* = 0.013; VN: *F*(2,45) = 3.364, *P* = 0.044, confirming significant change with age in all four networks. The rate of change of connectivity with age was greatest for the VN, as were the initial connectivity and the absolute increase in connectivity (black line in Fig. 4*A*). Note that visual connectivity increased rapidly in childhood, reaching a plateau by the beginning of adolescence. A permutation test compared the initial rate of change parameter between the VN (0.465) and the attentional networks (0.186). ROIs were collapsed across the three attentional networks for this analysis. The difference in the initial rate of change parameter was compared to a distribution computed under the null hypothesis that the network labels—visual or attentional—are interchangeable. That is, for each of 10,000 permutations ROIs were randomly reassigned to belong to either the visual (3 ROIs) or attentional (13 ROIs) networks, developmental trajectories were fitted for the two new networks, and the difference in the rate of change parameter between the two new networks was added to the null distribution. The permutation test indicated that the rate of change was significantly greater for the VN than the attentional networks (*P* = 0.022).

#### Between-network connectivity vs. age.

Figure 4*B* shows connectivity between each pair of cognitive networks (DMN-FPN, DMN-SN, FPN-SN); in each case, connectivity between the networks is positive and increases monotonically with age. The figure also shows the mean connectivity between the VN and the cognitive networks (COG-VIS; weighted to give equal weight to the 3 cognitive networks despite the differences in the number of ROIs). The between-network connectivity for the VN increased across childhood but reached a much lower value than that reached by the connectivity between the cognitive networks. The connectivity between the VN and the DMN reached a higher plateau than that between the VN and FPN or SN (data not shown); this may be because the precuneus, part of the DMN, is known to play a role as an extrastriate visual area (Stiers et al. 2006). Indeed, the precuneus, out of all the cognitive network areas, showed the strongest connectivity with the VN (Fig. 2).

The model again provided a significantly better fit than an intercept-only model in each case: DMN-FPN: *F*(2,45) = 10.485, *P* < 0.001; DMN-SN: *F*(2,45) = 8.272, *P* < 0.001; FPN-SN: *F*(2,45) = 6.413, *P* = 0.004; COG-VIS: *F*(2,45) = 4.939, *P* = 0.012. We also computed connectivity between the DMN and FPN after excluding the two DMN nodes in angular gyrus (left/right), which lie close to the FPN nodes in the intraparietal sulcus. Results were similar to when the angular gyrus nodes were included [data not shown; the model fit remained significant, *F*(2,45) = 9.074, *P* < 0.001, and the rate parameter increased very slightly to 0.200 from 0.196].

Thus our data confirm the prediction that positive correlation between nodes of the FPN and DMN and the SN and DMN increases with age in a similar manner to connectivity within these networks (mean initial rate parameter for growth of connectivity with age within the FPN, DMN, and SN is 0.20/yr, and mean parameter for growth of connectivity between these networks is 0.17/yr).

## DISCUSSION

We found that positive correlations between beta amplitude envelopes increased between nodes of the DMN, FPN, and SN throughout adolescence and into young adulthood. Furthermore, the increase in correlation strength between the three attentional networks (DMN, FPN, and SN) followed a trajectory similar to that of the correlations within these networks. In contrast to these slowly maturing correlations within and between attentional networks, we found that correlations between nodes of the VN matured rapidly during childhood.

Given that the nodes of the VN are confined to occipital cortex, whereas the FPN and DMN extend from parietal to frontal cortex, this finding is, in itself, consistent with the observation of Fair et al. that network maturation involves a shift from local to more long-range connectivity patterns. However, we also observed that the connections within the SN follow a developmental trajectory similar to those in FPN and DMN, maturing more slowly than those in the VN, despite the fact that the average distance between the nodes in the SN (47 mm) is similar to the average distance between sites in the VN (45 mm). This suggests that slower maturation is specific to connections with anterior brain regions rather than with greater distance between network nodes per se. This would be consistent with the extensive evidence that association cortex in the frontal lobe, involved in executive functions, is a late-developing region of the cortex (Fuster 2002).

The finding that beta amplitude correlations within the attentional networks followed a developmental trajectory similar to correlations between networks suggests that beta amplitude correlations reflect a process responsible for integration of activity within and between these networks. Attentional regulation depends on the capacity to switch off processing of situationally irrelevant information while prioritizing the processing of relevant information. Our findings are consistent with the expectation that the maturation of attentional control not only depends on the strengthening of communication within specialized attentional networks but also requires the strengthening of integration between these networks. This conclusion is similar to the conclusion of Marek et al. (2015) based on correlations between resting-state BOLD signals that the transition to adult-level inhibitory control is dependent upon the refinement and strengthening of integration between specialized networks. However, it should be noted that, consistent with much other data regarding BOLD signals, Marek et al. found that resting-state BOLD signal in nodes of the DMN is only minimally correlated with that in other resting-state networks including the FPN and SN.

Our finding of positive correlations in the beta band between the DMN and the other two attentional networks (FPN and SN) does not preclude the possibility that anticorrelation might be observed in other frequency bands. It is noteworthy that in a study employing intracerebral electrodes, Ossandón et al. (2011) observed transient suppression of power in the high gamma band (60–14 Hz) in the DMN during a visual search task. In contrast, there were increases in high gamma power in task-positive regions such as visual cortex and the dorsal attention network. There were widespread power decreases in beta-band power in DMN, visual cortex, and dorsal attention network. Thus, at least during that task, the relationship between beta and gamma oscillations differed between DMN and task-positive sites. Indeed, studies of intracerebral recordings in monkeys (Logothetis et al. 2001) and cats (Niessing et al. 2005) demonstrate that the BOLD signal is most closely related to gamma-band signals.

It is noteworthy that we observed that correlations between nodes of the VN were substantially stronger than correlations within the other three networks, whereas correlations between the VN and the other networks were appreciably lower than the correlations between the other three networks, especially in adults. This would be anticipated if functional connections within and between networks form, dissolve, and reform rapidly in such a manner that a particular node of one of the attentional networks is transiently engaged in communication not only with nodes within the same network but also with nodes from one of the other two networks. Thus the zero-order beta-band within-network correlations for each of the three attentional networks (DMN, FPN, SN) are likely to be reduced on account of the variance at each node that is shared with nodes from other networks. This explanation is supported by the analysis of dynamic beta-band connectivity in adult participants by de Pasquale et al. (2012). They computed pairwise correlations in a sliding window of 10-s duration. Within-network correlations in a particular network were appreciably greater during transient periods that de Pasquale et al. designated that region’s “maximum connectivity windows” (MCWs). Consistent with our findings, they found that during MCWs in which the DMN was maximally internally connected, the DMN was also appreciably more strongly correlated with other networks, especially the FPN, and a ventral attention network resembling the SN. On the other hand, during MCWs in which the visual network was maximally internally correlated, the visual network was only weakly correlated with other networks.

Our earlier investigation into the development of dynamic connectivity in the same participant sample (Brookes et al. 2018), identified eight transient spatio-temporal brain states with typical durations of order 50 ms. In the case of one of those transient states, which was characterized by high spatial loading in bilateral parietal and temporal cortex, the percentage of total time spent in the transient state showed a developmental trajectory similar to the development of connectivity within and between the FPN, DMN, and SN reported in this article (Fig. 4). Despite the marked differences in spatial distribution between that transient state and the three attentional networks examined in this article, the similarity in developmental trajectory raises the possibility that transient bursts of oscillations in parietal and temporal cortex might play a role in the coordination of activity within and between the FPN, DMN, and SN.

In interpreting our results several potential limitations should be borne in mind. Spurious enhancement of correlations between nodes due to leakage of signal between brain sites remains a possibility, although there were low correlations between beamformer weights for all pairs of network nodes and we took care to remove any zero-lag correlations before our connectivity analysis. The lower sensitivity of MEG to signals from deeper brain regions, together with our observation that as participants’ head sizes increased with age the signal strength recorded at the sensors from our ROIs increased, indicates that the effects we observed in the relatively deeply located SN must be regarded with caution. However, although we observed that controlling for variation in lead fields to correct for variation of head size did produce a slight reduction in the effects of age reported in this article, the adjustment was small compared with the observed effects of age.

Another caveat is that MEG may be less sensitive than fMRI at detecting changes in connectivity in short-range connections because of its lower spatial resolution. However, this is unlikely to account for our observation that beta-band connectivity between the long-range networks (DMN and FPN) developed at a rate similar to connectivity within these networks. MEG has higher temporal resolution than fMRI, allowing the investigation of signals in different frequency bands. Interestingly, Bastos et al. (2015) found that in primate visual areas feedforward signals were predominantly carried in the gamma and theta bands, whereas feedback signals were predominantly carried in the beta band. This lends support to the concept that beta-band signals play a higher-order role in coordinating activity within and between networks. Both within- and between-network connections would be expected to play a crucial role in well-coordinated, mature brain function. Our findings suggest that the development of beta-band connectivity plays a key role in the development of both within- and between-network coordination.

In summary, we have presented electrophysiological evidence that attentional networks develop throughout adolescence, continuing to develop after the primary visual network has matured at the end of childhood. Moreover, the synchrony of beta-band oscillatory power between the attentional networks increases at a rate similar to the synchrony within each network. The development of connectivity within and between attentional networks may contribute to the developing abilities of adolescents to focus their attention, exploit their growing working memory, and prioritize tasks and activities.

## GRANTS

P. M. Briley is a Royal College of Psychiatrists (RCPsych) Pathfinder Fellow and received funding toward this research project from RCPsych. H. J. F. Smith was supported by a Medical Research Council (MRC) studentship. G. L. Colclough was supported by the Research Councils UK Digital Economy Programme (EP/G036861/1, Centre for Doctoral Training in Healthcare Innovation). M. J. Brookes was supported by an MRC New Investigator Research Grant (MR/M006301/1). This work was also supported by an MRC Partnership Grant (MR/K005464/1) and an MRC grant (MR/J01186X/1). The Wellcome Centre for Integrative Neuroimaging is supported by core funding from the Wellcome Trust (203139/Z/16/Z).

## DISCLOSURES

No conflicts of interest, financial or otherwise, are declared by the authors.

## AUTHOR CONTRIBUTIONS

E.B.L., M.J.G., M.J.B., P.G.M., and P.F.L. conceived and designed research; H.J.F.S. performed experiments; P.M.B., E.B.L., H.J.F.S., and P.F.L. analyzed data; P.M.B., E.B.L., M.J.G., M.J.B., and P.F.L. interpreted results of experiments; P.M.B. prepared figures; P.M.B., E.B.L., and P.F.L. drafted manuscript; P.M.B., E.B.L., M.J.G., G.L.C., M.J.B., and P.F.L. edited and revised manuscript; P.M.B., E.B.L., M.J.G., G.L.C., M.J.B., and P.F.L. approved final version of manuscript.
